# A video analysis of clinical handovers between paramedics and emergency care staff

**DOI:** 10.29045/14784726.2019.06.4.1.44

**Published:** 2019-06-01

**Authors:** Ethan Shapiro

**Affiliations:** Northumbria University

## Abstract

**Aims:**

Studies examining communication during clinical handovers involving paramedics are rare. A recent National Institute of Health Research (NIHR) report has noted a significant gap in our knowledge and understanding of what happens during clinical handovers between ambulance services and emergency departments. This PhD study has used a video analysis to understand issues in communication and teamwork during these clinical handovers by examining cases of good practice.

**Methods:**

A video analysis of 100 examples of handovers from different TV programmes, including *24 Hours in A&E* and *The Real A&E*, was conducted. This methodology emphasised the capturing of real-world practice, without the limitation of semi-structured interviews which are reliant on recall of events. These examples highlighted how interprofessional teamworking and communication were conducted during these high pressured and complex situations. The videos were transcribed verbatim and idiosyncratically to show variances in the style of communication as well as the non-verbal actions used by the staff during the exchange of patient information.

**Results:**

The emergent findings demonstrate a variety of interactional strategies that paramedics use when conducting handovers. These strategies often involved a paramedic team member drawing attention to themselves by stating they were ready to do the handover or loudly introducing the patient’s name. This was typically followed by an account of the treatment the paramedic team provided and ended with questions from those receiving the handover. The questions asked by the receiving team would be about the treatment provided and during this exchange the paramedic team would justify the methods used in assisting the patient. When handovers were being taken for high-risk patients, for example ‘haemorrhaging of a femoral artery’, the structure of the handover changed. In such examples, the paramedic team were found to dictate how the receiving team handled continuation of treatment for the patient.

**Conclusion:**

The variations in handover examples that have been captured and analysed have been beneficial in understanding the interactions between paramedics and the emergency care team. Initial findings have shown how the structure of communication during handovers can be altered due to situational or contextual factors. These results have highlighted where potential improvements in communication can be made during handovers, which would subsequently increase patient safety and experience.

